# Open Targets Platform: supporting systematic drug–target identification and prioritisation

**DOI:** 10.1093/nar/gkaa1027

**Published:** 2020-11-16

**Authors:** David Ochoa, Andrew Hercules, Miguel Carmona, Daniel Suveges, Asier Gonzalez-Uriarte, Cinzia Malangone, Alfredo Miranda, Luca Fumis, Denise Carvalho-Silva, Michaela Spitzer, Jarrod Baker, Javier Ferrer, Arwa Raies, Olesya Razuvayevskaya, Adam Faulconbridge, Eirini Petsalaki, Prudence Mutowo, Sandra Machlitt-Northen, Gareth Peat, Elaine McAuley, Chuang Kee Ong, Edward Mountjoy, Maya Ghoussaini, Andrea Pierleoni, Eliseo Papa, Miguel Pignatelli, Gautier Koscielny, Mohd Karim, Jeremy Schwartzentruber, David G Hulcoop, Ian Dunham, Ellen M McDonagh

**Affiliations:** European Molecular Biology Laboratory, European Bioinformatics Institute (EMBL-EBI), Wellcome Genome Campus, Hinxton, Cambridgeshire CB10 1SD, UK; Open Targets, Wellcome Genome Campus, Hinxton, Cambridgeshire CB10 1SD, UK; European Molecular Biology Laboratory, European Bioinformatics Institute (EMBL-EBI), Wellcome Genome Campus, Hinxton, Cambridgeshire CB10 1SD, UK; Open Targets, Wellcome Genome Campus, Hinxton, Cambridgeshire CB10 1SD, UK; European Molecular Biology Laboratory, European Bioinformatics Institute (EMBL-EBI), Wellcome Genome Campus, Hinxton, Cambridgeshire CB10 1SD, UK; Open Targets, Wellcome Genome Campus, Hinxton, Cambridgeshire CB10 1SD, UK; European Molecular Biology Laboratory, European Bioinformatics Institute (EMBL-EBI), Wellcome Genome Campus, Hinxton, Cambridgeshire CB10 1SD, UK; Open Targets, Wellcome Genome Campus, Hinxton, Cambridgeshire CB10 1SD, UK; European Molecular Biology Laboratory, European Bioinformatics Institute (EMBL-EBI), Wellcome Genome Campus, Hinxton, Cambridgeshire CB10 1SD, UK; Open Targets, Wellcome Genome Campus, Hinxton, Cambridgeshire CB10 1SD, UK; European Molecular Biology Laboratory, European Bioinformatics Institute (EMBL-EBI), Wellcome Genome Campus, Hinxton, Cambridgeshire CB10 1SD, UK; Open Targets, Wellcome Genome Campus, Hinxton, Cambridgeshire CB10 1SD, UK; European Molecular Biology Laboratory, European Bioinformatics Institute (EMBL-EBI), Wellcome Genome Campus, Hinxton, Cambridgeshire CB10 1SD, UK; Open Targets, Wellcome Genome Campus, Hinxton, Cambridgeshire CB10 1SD, UK; European Molecular Biology Laboratory, European Bioinformatics Institute (EMBL-EBI), Wellcome Genome Campus, Hinxton, Cambridgeshire CB10 1SD, UK; Open Targets, Wellcome Genome Campus, Hinxton, Cambridgeshire CB10 1SD, UK; European Molecular Biology Laboratory, European Bioinformatics Institute (EMBL-EBI), Wellcome Genome Campus, Hinxton, Cambridgeshire CB10 1SD, UK; Open Targets, Wellcome Genome Campus, Hinxton, Cambridgeshire CB10 1SD, UK; European Molecular Biology Laboratory, European Bioinformatics Institute (EMBL-EBI), Wellcome Genome Campus, Hinxton, Cambridgeshire CB10 1SD, UK; Open Targets, Wellcome Genome Campus, Hinxton, Cambridgeshire CB10 1SD, UK; European Molecular Biology Laboratory, European Bioinformatics Institute (EMBL-EBI), Wellcome Genome Campus, Hinxton, Cambridgeshire CB10 1SD, UK; Open Targets, Wellcome Genome Campus, Hinxton, Cambridgeshire CB10 1SD, UK; European Molecular Biology Laboratory, European Bioinformatics Institute (EMBL-EBI), Wellcome Genome Campus, Hinxton, Cambridgeshire CB10 1SD, UK; Open Targets, Wellcome Genome Campus, Hinxton, Cambridgeshire CB10 1SD, UK; European Molecular Biology Laboratory, European Bioinformatics Institute (EMBL-EBI), Wellcome Genome Campus, Hinxton, Cambridgeshire CB10 1SD, UK; Open Targets, Wellcome Genome Campus, Hinxton, Cambridgeshire CB10 1SD, UK; European Molecular Biology Laboratory, European Bioinformatics Institute (EMBL-EBI), Wellcome Genome Campus, Hinxton, Cambridgeshire CB10 1SD, UK; Open Targets, Wellcome Genome Campus, Hinxton, Cambridgeshire CB10 1SD, UK; European Molecular Biology Laboratory, European Bioinformatics Institute (EMBL-EBI), Wellcome Genome Campus, Hinxton, Cambridgeshire CB10 1SD, UK; Open Targets, Wellcome Genome Campus, Hinxton, Cambridgeshire CB10 1SD, UK; European Molecular Biology Laboratory, European Bioinformatics Institute (EMBL-EBI), Wellcome Genome Campus, Hinxton, Cambridgeshire CB10 1SD, UK; Open Targets, Wellcome Genome Campus, Hinxton, Cambridgeshire CB10 1SD, UK; Open Targets, Wellcome Genome Campus, Hinxton, Cambridgeshire CB10 1SD, UK; GlaxoSmithKline plc, GSK Medicines Research Centre, Gunnels Wood Road, Stevenage SG1 2NY, UK; Open Targets, Wellcome Genome Campus, Hinxton, Cambridgeshire CB10 1SD, UK; GlaxoSmithKline plc, GSK Medicines Research Centre, Gunnels Wood Road, Stevenage SG1 2NY, UK; European Molecular Biology Laboratory, European Bioinformatics Institute (EMBL-EBI), Wellcome Genome Campus, Hinxton, Cambridgeshire CB10 1SD, UK; Open Targets, Wellcome Genome Campus, Hinxton, Cambridgeshire CB10 1SD, UK; European Molecular Biology Laboratory, European Bioinformatics Institute (EMBL-EBI), Wellcome Genome Campus, Hinxton, Cambridgeshire CB10 1SD, UK; Open Targets, Wellcome Genome Campus, Hinxton, Cambridgeshire CB10 1SD, UK; European Molecular Biology Laboratory, European Bioinformatics Institute (EMBL-EBI), Wellcome Genome Campus, Hinxton, Cambridgeshire CB10 1SD, UK; Open Targets, Wellcome Genome Campus, Hinxton, Cambridgeshire CB10 1SD, UK; Open Targets, Wellcome Genome Campus, Hinxton, Cambridgeshire CB10 1SD, UK; Wellcome Sanger Institute, Wellcome Genome Campus, Hinxton, Cambridgeshire CB10 1SA, UK; Open Targets, Wellcome Genome Campus, Hinxton, Cambridgeshire CB10 1SD, UK; Wellcome Sanger Institute, Wellcome Genome Campus, Hinxton, Cambridgeshire CB10 1SA, UK; European Molecular Biology Laboratory, European Bioinformatics Institute (EMBL-EBI), Wellcome Genome Campus, Hinxton, Cambridgeshire CB10 1SD, UK; Open Targets, Wellcome Genome Campus, Hinxton, Cambridgeshire CB10 1SD, UK; Open Targets, Wellcome Genome Campus, Hinxton, Cambridgeshire CB10 1SD, UK; Systems Biology, Biogen, Cambridge, MA 02142, USA; European Molecular Biology Laboratory, European Bioinformatics Institute (EMBL-EBI), Wellcome Genome Campus, Hinxton, Cambridgeshire CB10 1SD, UK; Open Targets, Wellcome Genome Campus, Hinxton, Cambridgeshire CB10 1SD, UK; Open Targets, Wellcome Genome Campus, Hinxton, Cambridgeshire CB10 1SD, UK; GlaxoSmithKline plc, GSK Medicines Research Centre, Gunnels Wood Road, Stevenage SG1 2NY, UK; Open Targets, Wellcome Genome Campus, Hinxton, Cambridgeshire CB10 1SD, UK; Wellcome Sanger Institute, Wellcome Genome Campus, Hinxton, Cambridgeshire CB10 1SA, UK; Open Targets, Wellcome Genome Campus, Hinxton, Cambridgeshire CB10 1SD, UK; Wellcome Sanger Institute, Wellcome Genome Campus, Hinxton, Cambridgeshire CB10 1SA, UK; Open Targets, Wellcome Genome Campus, Hinxton, Cambridgeshire CB10 1SD, UK; GlaxoSmithKline plc, GSK Medicines Research Centre, Gunnels Wood Road, Stevenage SG1 2NY, UK; European Molecular Biology Laboratory, European Bioinformatics Institute (EMBL-EBI), Wellcome Genome Campus, Hinxton, Cambridgeshire CB10 1SD, UK; Open Targets, Wellcome Genome Campus, Hinxton, Cambridgeshire CB10 1SD, UK; Wellcome Sanger Institute, Wellcome Genome Campus, Hinxton, Cambridgeshire CB10 1SA, UK; European Molecular Biology Laboratory, European Bioinformatics Institute (EMBL-EBI), Wellcome Genome Campus, Hinxton, Cambridgeshire CB10 1SD, UK; Open Targets, Wellcome Genome Campus, Hinxton, Cambridgeshire CB10 1SD, UK

## Abstract

The Open Targets Platform (https://www.targetvalidation.org/) provides users with a queryable knowledgebase and user interface to aid systematic target identification and prioritisation for drug discovery based upon underlying evidence. It is publicly available and the underlying code is open source. Since our last update two years ago, we have had 10 releases to maintain and continuously improve evidence for target–disease relationships from 20 different data sources. In addition, we have integrated new evidence from key datasets, including prioritised targets identified from genome-wide CRISPR knockout screens in 300 cancer models (Project Score), and GWAS/UK BioBank statistical genetic analysis evidence from the Open Targets Genetics Portal. We have evolved our evidence scoring framework to improve target identification. To aid the prioritisation of targets and inform on the potential impact of modulating a given target, we have added evaluation of post-marketing adverse drug reactions and new curated information on target tractability and safety. We have also developed the user interface and backend technologies to improve performance and usability. In this article, we describe the latest enhancements to the Platform, to address the fundamental challenge that developing effective and safe drugs is difficult and expensive.

## INTRODUCTION

The drug discovery and development process is costly and ineffective; it is predicted that around 90% of drugs entering phase 1 clinical trials will not reach approval, and overall costs for each approved compound come to around $1.4 billion (1,2). In addition, patients treated with approved drugs may experience a lack of therapeutic response or adverse drug reactions, and many diseases still remain untreatable. The aim of the Open Targets consortium, which brings together research institutes, academic and industry partners in a pre-competitive collaboration, is to address the fundamental issue of drug attrition due to a lack of efficacy or safety, and support the identification of novel targets for disease treatment. The Open Targets Platform (https://www.targetvalidation.org/) provides an open source, publicly available knowledgebase and tools that enable evidence-based systematic prioritisation of targets for disease treatment (3,4). Our informatics pipeline addresses the challenges of ingesting different datasets and formats, handles large amounts of data, and standardises the data to integrate it together into one platform.

In the past two years, we have built upon this foundation by expanding target–disease evidence data, adding pharmacovigilance, safety and tractability information, improving the scoring of evidence and prioritisation of targets, and enriching our disease ontology. We have incorporated a new drug index to include all parent molecules with known pharmacological action or disease indication. We have integrated novel data generated from Open Targets consortium informatics and experimental projects. These updates have been informed by our users and members of the Open Targets consortium. We have also expanded our training and outreach scope, providing tutorials and interactive sessions to help inform and support users.

Users can explore therapeutic hypotheses within the Platform, ensuring that targets have supportive evidence for efficacy and safety prior to transition to the next stages of drug development. These preclinical target assessments are important as they can increase the chance of drug approval for specific indications. For example, drugs that have targets with underlying evidence for a genetic association with the relevant disease are twice as likely to succeed in clinical trials and be approved (5,6). To this end, a major new feature in the Platform is the incorporation of evidence from the Open Targets Genetics Portal (https://genetics.opentargets.org/), which integrates publicly available human genome-wide association (GWAS) data with functional genomics to associate disease loci with target genes (7). The evidence from the genetics portal is integrated into our scoring system and informs target prioritisation for a given disease. With regard to safety, a key addition we provide is the evaluation of significant post-marketing adverse drug reactions from the FDA Adverse Event Reporting System (FAERS) for approved drugs, as well as curated safety information, to help inform the potential impact of modulating a given target.

Herein, we detail the key enhancements to the data and features within the Open Targets Platform, as well as improvements to the user interface and underlying technology.

### Revisiting the open targets platform

**Figure 1.**
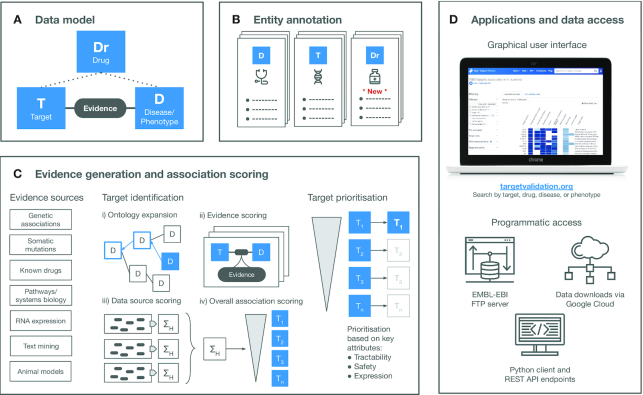
Overview of the Open Targets Platform. (**A**) The Platform data model includes the entities targets, diseases and drugs. The relationships between the three entities is shown. (**B**) Annotation of biomedical entities is provided from 26 underlying data sources. (**C**) The target identification and prioritisation framework is based on evidence from 20 evidence sources providing target–disease relationships. EFO expansion allows for capture of further associations between targets and diseases/phenotypes. For each target–disease association, the underlying data sources that provide evidence are scored, and an overall scoring ranks targets associated with the disease. Targets are further prioritised based on additional key attributes including tractability, safety and expression. (**D**) Platform data is accessible via a user interface or programmatically via the EMBL-EBI FTP server, API endpoints or as downloads via Google Cloud. Abbreviations: D, disease/phenotype; Dr, drug; EFO, Experimental factor ontology; T, target.

Figure 1 provides a visual overview of the Open Targets Platform; the underlying data model representation (Figure 1A), entity details (Figure 1B), evidence generation and target–disease association scoring to aid target prioritisation (Figure 1C). The data and analyses are available through the user interface and programmatically (Figure 1D). Each stage is described in more detail below, with updates since our last publication (3).

### A universe of data built around targets, diseases and drugs

The decision-making process in a drug discovery project requires a thorough understanding of as many variables as possible to maximise the clinical trial success. The Open Targets Platform, therefore, aims to provide a comprehensive characterisation of targets, diseases or phenotypes and, more recently, known drugs that can help inform target identification and prioritisation (Figure 1B). To reconstruct these main biomedical concepts, we retrieve information from 26 different data sources (Supplementary Table S1). While most datasets are seamlessly integrated, others require some post-processing. For example, our focus on drug targets implies that all gene products could potentially be targeted, so information from core resources such as Ensembl (8) or Uniprot (9) needs to be integrated to cover both RNAs and proteins. Sometimes, more detailed analysis is required to extract the relevant information or adjust the available data to a clinical setup. To recapitulate all the literature available for each of the entities, for example, we performed named-entity recognition on the available abstracts from Europe PMC (https://link.opentargets.io/). Other recent additions, such as the chemical probes or the target enabling packages require a consistent manual curation effort as data is scattered across different resources (10–12).

**Figure 2.**
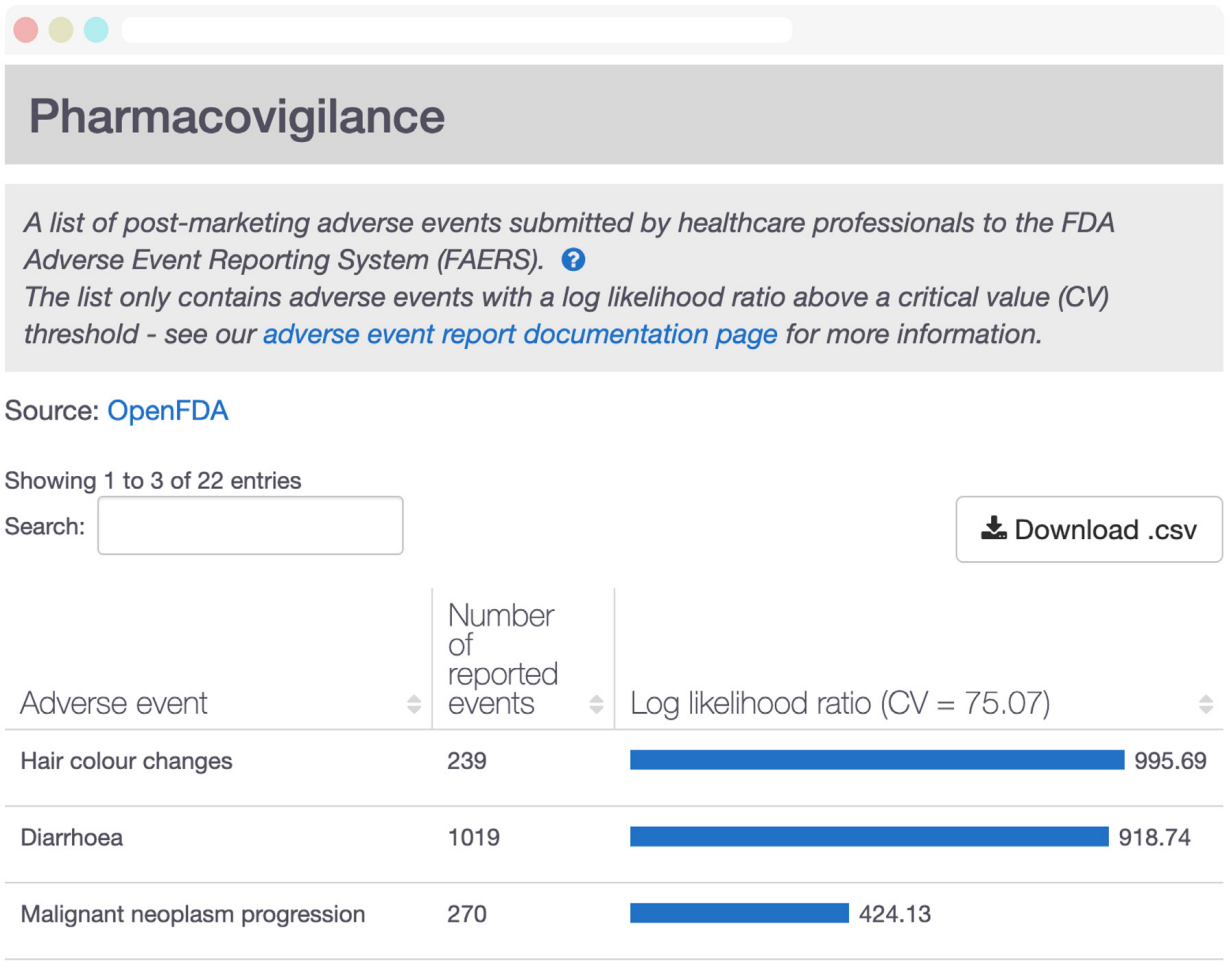
Post-marketing pharmacovigilance analysis for Pazopanib. Significant adverse events associated with Pazopanib (CHEMBL477772), based on systematic analysis of all available FDA Adverse Event Reporting System. Analysis is displayed in the Open Targets Platform for all drugs with available data.

To provide a more complete representation of the therapeutic space, we recently expanded our entities to include drugs from the ChEMBL database (13). ChEMBL curates and aggregates bioactive molecules with drug-like properties, as well as records from different public resources including Drugs@FDA, ClinicalTrials.gov and DailyMed, among others. The new drug index consists of all parent molecules with known pharmacological action or disease indication to a total of 6515 entries belonging to seven different modalities. Among the most relevant drug information, users can find curated mechanisms of action, approved or experimental indications, small molecule representations, synonyms and trade names. Moreover, we expanded the drug annotation with a statistical analysis on post-marketing significant adverse drug reactions (ADRs) from FDA Adverse Event Reporting System (FAERS) (14). From the >12 million publicly available reports in FAERS, we filtered the most reliable entries following similar published approaches (15). The significant drug-ADR pairs were then evaluated using a Likelihood Ratio Test (LRT) and critical values inferred using a Montecarlo simulation (16). The significance of a given drug-ADR is implicitly corrected by how often a drug is found in a report and how often an event is reported across drugs (Figure 2).

### Continuous improvement of target–disease evidence

**Figure 3.**
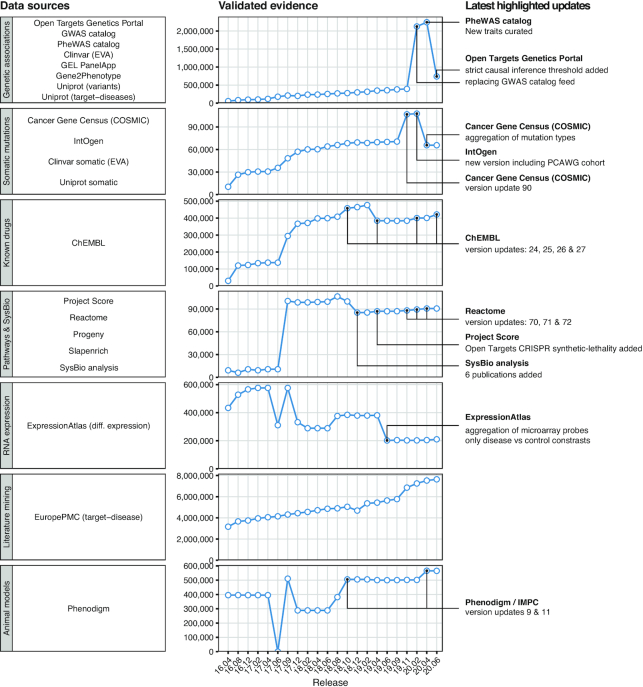
Target - disease evidence in the Open Targets Platform. Data sources are grouped by data type—left. Unique validated evidence available in each of the platform releases since April 2016 (16.04)—middle. Relevant changes in the most recent period are annotated in further detail—right. A full list of the data sources with references is found in Supplementary Table S2.

Identifying evidence implicating targets with diseases or phenotypes constitutes one of the pivotal challenges of the Open Targets Platform (Figure 1C). We currently maintain 20 different data sources capturing knowledge on target–disease relationships covering the following categories: genetic associations (for germline variation on common and rare diseases), somatic mutations, drugs, pathways and systems biology, RNA expression, text mining and animal models (Figure 3). All 10 154 924 pieces of evidence are mapped and curated using a reference target entity identifier (Ensembl gene) and disease or phenotype identifier (experimental factor ontology, EFO) (17).

The nature of the data sources vary and therefore have different requirements to keep them updated. Many remain stable and provide constant updates for every one of our releases—such as ChEMBL (13), COSMIC (18), Reactome (19), ExpressionAtlas (20), Cancer Gene Census (21) or ePMC (22). Some other providers (such as IntOgen) undergo major upgrades that require changes to our data model or considerable manual curation effort to integrate. During 2020, we have also included the curation of COVID-19 evidence, for example the 299 clinical trials with COVID-19 as primary indication and known drug targets annotated by ChEMBL. The most important changes to our evidence throughout the most recent period are shown in Figure 3. Supplementary Figure S1 provides an overview of the number of validated evidence strings from each individual source for each Platform release.

### Project score

In April 2019, we expanded our range of evidence to include the genome-wide CRISPR–Cas9 dropout screens conducted by Behan *et al.* (23). This collaborative Open Targets study performed 941 fitness screens in 339 cancer cell lines targeting 18 009 genes. Moreover, a prioritisation framework designated ‘Project Score’ was developed to integrate cell fitness effects, genomic biomarkers and target tractability, to systematically prioritise new cancer targets (https://score.depmap.sanger.ac.uk/). A minimum target priority score of 40 is used (based on scores calculated for targets with approved or preclinical cancer compounds), providing a dataset of synthetic-lethality evidence for the association between 623 genes and 19 cancer types (Supplementary Figure S1).

### Open targets genetics portal

Genome-wide association studies (GWAS) provide a rich source of disease-associated genomic loci. Nevertheless, it remains a long-standing challenge to link these loci to targetable causal genes. The Open Targets Genetics Portal addresses this problem by interpreting manually curated associations from the GWAS catalog, as well as independent signals from GWAS with publicly available summary statistics, most importantly the UK Biobank GWAS data (7,24,25). The Genetics Portal performs fine-mapping to narrow down the likely set of causal variants at a given trait-associated locus and to identify the potential causal gene for a particular association. The recently added locus-to-gene score (L2G) uses machine learning to prioritise causal genes by integrating fine-mapping credible sets, QTL colocalisation and functional genomics data. This method can pinpoint causal connections between loci and distant genes, and can predict multiple causal genes, a significant improvement over approaches based on gene distance to lead SNPs.

The inclusion of the Genetics Portal evidence in the Open Targets Platform supersedes the previous GWAS catalog evidence, removed in release 20.02 (Figure 3, Supplementary Figure S1). The inclusion of a more stringent GWAS p-value cutoff (5e–8 instead of 1e–5) removed 69 298 non-significant GWAS catalog evidence data points. Overall, the Genetics Portal provides a cutting-edge framework to obtain the most up-to-date GWAS evidence for complex or common diseases or phenotypes.

### Evolving target identification and prioritisation

A few challenges remain after evidence is appropriately collected and normalised (Figure 1C):

1. **Annotation sparsity**. Evidence can sometimes be informative to discriminate targets in similar diseases or phenotypes. For example, a target associated with Crohn's disease could also be associated, albeit indirectly, with the more general term ‘inflammatory bowel disease’. To systematically benefit from this evidence, we take advantage of the ontological properties of EFO by propagating evidence from any child node to its parent nodes all the way up to its corresponding therapeutic area(s) (Figure 1C, i). In the 19.11 release, we adopted EFO v3 which transformed our reference ontology to include other existing domain-specific ontologies. By liaising with the Monarch Disease Ontology (MonDO) (26), EFO v3 provides a comprehensive ontology of diseases and phenotypes as well as cutting-edge algorithmic and manual curation to classify them using different ontologies. To better align with the clinical purpose of the Open Targets Platform, we collaborated to reorganise the EFO diseases using the most relevant therapeutic areas. As a result of adopting EFO v3, the number of associations increased by 90% in the 19.11 release.
2. **Target–disease evidence scoring**. Deciding what constitutes a strong evidence source and how it compares with similar data sources remains an ongoing question and is open for interpretation. The lack of appropriate gold standards across therapeutic areas limits the effectiveness of appropriate benchmarks. Despite these limitations, we score all our evidence in the range between 0 and 1, providing an informed estimation on the strength of the association between the target and the disease (Figure 1C, ii). The scoring functions for all data source evidence including the latest modifications are listed in Supplementary Table S2. This is regularly reviewed and benchmarked, in particular when new datasets are introduced.
3. **Consolidating evidence into target–disease associations at data source or data type level**. Independent evidence for the same target–disease pair might accumulate boosting the confidence in that particular association. The platform attempts to group the repeated evidence per data source by calculating the harmonic sum of the vector of evidence scores (Figure 1C, iii). Moreover, to provide an indicative score for groupings of data sources based on the nature of the evidence (e.g. genetic association, known drugs), a weighted harmonic sum is estimated for each of the data types (3).
4. **Estimating an overall association score**. To summarize the overall strength of a given target–disease association, we perform a weighted harmonic sum of the association scores using all individual data-source specific scores (Figure 1C iv). The resulting overall score is provided for each of the 7 282 832 target–disease associations. All weights are listed in Supplementary Table S2 (weight factor), including the most recent changes (3).

By following this multi-step process, the Open Targets Platform presents a ranked list of the targets associated with a disease or phenotype or vice versa. However, the prioritisation of targets sometimes requires the addition of extra information on how suitable these targets are for a given therapeutic hypothesis, for example how tractable the target is to modulation by different drug modalities (Figure 1C). To expand on target annotations that can assist in decision-making when reviewing a target list, an updated Open Targets tractability assessment for small molecules or antibodies is now included on our prioritisation view (27). We also provide information on target safety where available, including known side effects (28,29), safety risk information (30,31) and non-clinical experimental toxicity (32,33).

### Enhanced interface and upcoming changes

Recognising the need to deliver a best-in-class user experience, we commenced a two-year project to redesign the Open Targets Platform and its technical infrastructure. The project emerged from user feedback that identified new requirements to streamline the overall user journey, enhance entity annotations, and support the exploration of different therapeutic hypotheses. External contributors also require a more amenable codebase to expand the current functionalities on their own private instances. Further, our web analytics pointed to increasing interest in specific sections, including target tractability and safety. Overall, a modern technical infrastructure combined with a fresh new interface will ensure the Platform can continue to adapt to more complex data and generate further unique insights in the drug discovery area.

**Figure 4.**
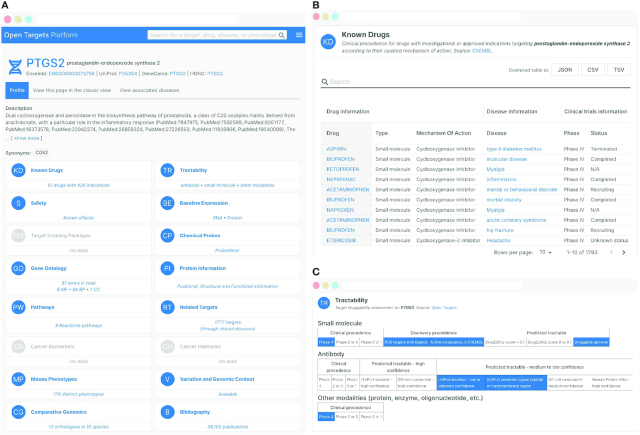
Enhanced user interface and new functionality. Platform interface redesign for *PTGS2* target profile page. (**A**) Identifiers and links to other resources are provided at the top of the page. Target information widgets outline the available data (in blue). By clicking on a widget, the user is taken to that section. Sections can be rearranged by the user, allowing them to personalise their experience. (**B**) The ‘Known drugs’ widget takes the user to a sortable and filterable table including information on clinical candidates or approved drugs, sourced from ChEMBL (12). (**C**) The ‘Tractability’ section provides a druggability assessment by small molecule, antibody or other modality.

In the redesigned version of the Platform, users have access to a powerful search functionality that includes the ability to search by drug, trade name and generic names and synonyms. Updated entity profile pages contain summary widgets that provide an at-a-glance overview of the data available for a specific entity (Figure 4). A scrollable page with detailed views provides more in-depth aggregation and analysis of data. Users can also rearrange and reorder summary widgets and detail views to customise their experience based on data they frequently access. Whilst this new version of the Platform is still under active development, users are able to move between the current version of the Platform and the new redesigned Platform. In addition to an enhanced user interface, the redesigned Platform also includes a new GraphQL API that allows for more powerful and nuanced queries of the data.

### A technical look into Open Targets Platform

All data in the Open Targets Platform is publicly available. However some datasets require ad hoc pipelines to perform mapping or post-processing steps required to align them to the current data model. A JSON validator ensures all evidence respects the schema. All annotation, evidence and ontologies are then processed by the data pipeline to reconstruct entities, clean and score evidence and associations, generate search indexes and calculate disease-disease similarities. The extract-transform-load pipeline is written in Python v3 and the results loaded in ElasticSearch v7.6. The REST API is currently available at https://platform-api.opentargets.io/ and implemented using Python v2 and Flask framework v1.1. The web application is written using Angular 1.7 as well as a number of libraries such as D3.js v.3.5 for interactive visualisations. To ensure global access, the infrastructure is deployed across three different regions. Each regional deployment is the same and globally balanced. All our services are configured, loaded and optimised in Google Cloud Platform and our code is open source and accessible in the repositories listed in Supplementary Table S3.

Our pipeline and infrastructure is currently undergoing the aforementioned re-design towards more modern programming languages (Scala v2.12), frameworks (Apache Spark v3.0.0, React 16.8, Sangria v2.0) and technologies (Elasticsearch v7.6, Clickhouse v20.5). Partially released during 2020, these technologies will enable new functionalities in the Open Targets Platform.

### Data availability and outreach activities

The Open Targets Platform is publicly available at https://www.targetvalidation.org/ and there are five releases each year that include updates to existing annotation and evidence data and integration of new datasets and features based on user requests and scientific advancements. Details of each release are available from the Open Targets blog and the Platform release and technical notes. The output data from our pipelines remains accessible in different formats depending on the user's individual requirements. It is available through the user interface, various REST API endpoints, our Python client, and our Data Downloads page. All data, which includes the input files used for each release pipeline run, can also be downloaded from EMBL-EBI’s FTP service (Figure 1D).

To support general and more disease-specific use cases of the Platform, we continue to offer free hands-on workshops on how to use the Open Targets Platform in webinar and face-to-face formats (34). Over the past two years, we have delivered training workshops in the United Kingdom, United States of America, Saudia Arabia, South Korea and Romania. Furthermore, given our commitment to open source software and open science, our entire codebase, including data integration and analysis pipelines and user interface, is available on GitHub and licensed under the Apache License Version 2.0. A full list of relevant data access, availability, and outreach resources are provided in Supplementary Table S4.

## DISCUSSION

The drug discovery process remains a complex challenge in which systematic data integration can help unravel new findings. Although progress has been made on improving data standards (for example through efforts such as the Global Alliance for Genomics and Health (https://www.ga4gh.org/genomic-data-toolkit/), Elixir (https://elixir-europe.org/services/tag/interoperability-and-standards), and the Gene Curation Coalition https://thegencc.org/), harmonizing different biomedical datasets still constitutes one of the key bottlenecks when it comes to interpreting the available knowledge. Several other resources including Pharos (35), Disgenet (36) or CanSAR (37) have recently approached the target prioritisation problem, providing a complementary view given their respective areas of expertise (38). The Open Targets Platform aims to help address this challenge by providing users with an up-to-date systematic interpretation of the relevant resources across therapeutic areas and—ultimately—complete the knowledge-gap with data generated within the Open Targets consortium.

By expanding the Open Targets Platform to new entities such as drugs, we hope to enhance the ability for our data model to capture information in order to better answer real world questions. This has allowed us to incorporate important drug and compound information such as mode of action, approved and experimental indications, small molecule representations as well as analysis of post-marketing ADRs. Enhanced target information includes chemical probe and target enabling packages information. Adoption of EFO version 3 has been pivotal in expanding the number of target–disease associations within the Platform. The expanded ontology allows new direct target–disease associations to be incorporated from the data sources, as well as ‘indirect’ associations where evidence is applied throughout the ontological structure of disease classification. As an example, this can be useful to users interested in targets that have been associated with a broad therapeutic area, or conversely a very specific disease. It also allows the collation of evidence for a target for a given therapeutic area, when the evidence annotations may be sparse for individual diseases that fall under this.

We have continued to update evidence for target–disease associations through routine releases from our data providers, as well as additional experimental evidence generated through Open Targets projects (such as Project Score) and the statistical genetics analyses from the Open Targets Genetics Portal. This has included clinical trial and drug target information for COVID-19 to aid in the effort to identify targets for the repurposing of existing drugs or development of novel treatments to help fight the pandemic. We have reviewed and reiterated our scoring of evidence, and enhanced the prioritisation of targets for particular diseases with the addition of tractability and safety information generated through Open Targets informatics projects. We are implementing a redesign of the Portal infrastructure, data pipelines and frontend to enhance usability and allow for more complexity in the data for the future.

The emergence of new large-scale technologies with increased evidence granularity (e.g. scRNAseq or CRISPR), as well as the increasing interest to stratify diseases based on these or other phenotypic readouts, introduces challenges that the Platform will seek to address in the near future through adaptation of our data model and new ways to represent data. Determining the relative importance of different pieces of evidence when combining the available information to suggest potentially successful targets for drug discovery is a further challenge. The inclusion of new systematic data sources, such as the state-of-the-art GWAS data from Open Targets Genetics Portal, revealed the need to appropriately weight and benchmark scored evidence against other orthogonal data sources, a challenging task due to the lack of appropriate gold standards. Moreover, recent studies have pinpointed to the usefulness of expanding experimentally determined evidence using protein interaction networks (39,40). Network data can help to circumvent issues such as non-tractable targets or safety liabilities, as well as identify functionally linked novel targets with no prior evidence. We are therefore exploring different approaches to further exploit the Platform target–disease evidence in the context of their molecular interactions.

To address a diverse set of challenges, and to ensure that the data within the Platform remains at the cutting-edge to inform drug discovery decision-making, we will work alongside our data providers and the Open Targets consortium members to introduce innovative solutions for the systematic identification and prioritisation of targets based on diverse and complex publicly available data.

## Supplementary Material

gkaa1027_Supplemental_File

## Notes

Present address: Ellen M. McDonagh, Open Targets and European Molecular Biology Laboratory-European Bioinformatics Institute (EMBL-EBI), Wellcome Genome Campus, Hinxton, Cambridgeshire CB10 1SD, UK.

Present address: David Ochoa, Open Targets and European Molecular Biology Laboratory-European Bioinformatics Institute (EMBL-EBI), Wellcome Genome Campus, Hinxton, Cambridgeshire CB10 1SD, UK.

## References

[1] Dimasi J.A., Grabowski H.G., Hansen R.W. Innovation in the pharmaceutical industry: new estimates of R&D costs. J. Health Econ. 2016; 47:20–33. 26928437 10.1016/j.jhealeco.2016.01.012

[2] Hay M., Thomas D.W., Craighead J.L., Economides C., Rosenthal J. Clinical development success rates for investigational drugs. Nat. Biotechnol. 2014; 32:40–51. 24406927 10.1038/nbt.2786

[3] Carvalho-Silva D., Pierleoni A., Pignatelli M., Ong C., Fumis L., Karamanis N., Carmona M., Faulconbridge A., Hercules A., McAuley E. et al. Open Targets Platform: new developments and updates two years on. Nucleic Acids Res. 2019; 47:D1056–D1065. 30462303 10.1093/nar/gky1133 PMC6324073

[4] Koscielny G., An P., Carvalho-Silva D., Cham J.A., Fumis L., Gasparyan R., Hasan S., Karamanis N., Maguire M., Papa E. et al. Open Targets: a platform for therapeutic target identification and validation. Nucleic Acids Res. 2017; 45:D985–D994. 27899665 10.1093/nar/gkw1055 PMC5210543

[5] King E.A., Davis J.W., Degner J.F. Are drug targets with genetic support twice as likely to be approved? Revised estimates of the impact of genetic support for drug mechanisms on the probability of drug approval. PLoS Genet. 2019; 15:e1008489. 31830040 10.1371/journal.pgen.1008489 PMC6907751

[6] Nelson M.R., Tipney H., Painter J.L., Shen J., Nicoletti P., Shen Y., Floratos A., Sham P.C., Li M.J., Wang J. et al. The support of human genetic evidence for approved drug indications. Nat. Genet. 2015; 47:856–860. 26121088 10.1038/ng.3314

[7] Ghoussaini M., Mountjoy E., Carmona M., Peat G., Ellen Hercules A., Fumis L., Miranda A., Carvalho-Silva D., Buniello A. et al. Open Targets Genetics: systematic identification of trait-associated genes using large-scale genetics and functional genomics. Nucleic Acids Res. 2020; doi:10.1093/nar/gkaa840. 10.1093/nar/gkaa840 PMC7778936 33045747

[8] Yates A.D., Achuthan P., Akanni W., Allen J., Allen J., Alvarez-Jarreta J., Amode M.R., Armean I.M., Azov A.G., Bennett R. et al. Ensembl 2020. Nucleic Acids Res. 2019; 48:D682–D688. 10.1093/nar/gkz966 PMC7145704 31691826

[9] Consortium U. UniProt: a worldwide hub of protein knowledge. Nucleic Acids Res. 2019; 47:D506–D515. 30395287 10.1093/nar/gky1049 PMC6323992

[10] Arrowsmith C.H., Audia J.E., Austin C., Baell J., Bennett J., Blagg J., Bountra C., Brennan P.E., Brown P.J., Bunnage M.E. et al. The promise and peril of chemical probes. Nat. Chem. Biol. 2015; 11:536–541. 26196764 10.1038/nchembio.1867 PMC4706458

[11] Jones M.M., Castle-Clarke S., Brooker D., Nason E., Huzair F., Chataway J. The structural genomics consortium: a knowledge platform for drug discovery: a summary. Rand Health Q. 2014; 4:19. 28560088 10.7249/rhq-04-03-19 PMC5396214

[12] Müller S., Ackloo S., Arrowsmith C.H., Bauser M., Baryza J.L., Blagg J., Böttcher J., Bountra C., Brown P.J., Bunnage M.E. et al. Donated chemical probes for open science. eLife. 2018; 7:e34311. 29676732 10.7554/eLife.34311 PMC5910019

[13] Mendez D., Gaulton A., Bento A.P., Chambers J., Marleen Félix E., María Juan Mutowo P., Nowotka M. et al. ChEMBL: towards direct deposition of bioassay data. Nucleic Acids Res. 2019; 47:D930–D940. 30398643 10.1093/nar/gky1075 PMC6323927

[14] Kumar A. The newly available FAERS public dashboard: implications for health care professionals. Hosp. Pharm. 2018; 54:75–77. 30923396 10.1177/0018578718795271 PMC6431724

[15] Maciejewski M., Lounkine E., Whitebread S., Farmer P., DuMouchel W., Shoichet B.K., Urban L. Reverse translation of adverse event reports paves the way for de-risking preclinical off-targets. eLife. 2017; 6:e25818. 28786378 10.7554/eLife.25818 PMC5548487

[16] Huang L., Zalkikar J., Tiwari R.C. Likelihood ratio test-based method for signal detection in drug classes using FDA’s AERS database. J. Biopharm. Stat. 2013; 23:178–200. 23331230 10.1080/10543406.2013.736810

[17] Malone J., Holloway E., Adamusiak T., Kapushesky M., Zheng J., Kolesnikov N., Zhukova A., Brazma A., Parkinson H. Modeling sample variables with an experimental factor ontology. 2010; 26:1112–1118. 10.1093/bioinformatics/btq099 PMC2853691 20200009

[18] Tate J.G., Bamford S., Jubb H.C., Sondka Z., Beare D.M., Bindal N., Boutselakis H., Cole C.G., Creatore C., Dawson E. et al. COSMIC: the catalogue of somatic mutations in cancer. Nucleic Acids Res. 2019; 47:D941–D947. 30371878 10.1093/nar/gky1015 PMC6323903

[19] Jassal B., Matthews L., Viteri G., Gong C., Lorente P., Fabregat A., Sidiropoulos K., Cook J., Gillespie M., Haw R. et al. The reactome pathway knowledgebase. Nucleic Acids Res. 2020; 48:D498–D503. 31691815 10.1093/nar/gkz1031 PMC7145712

[20] Papatheodorou I., Moreno P., Manning J., Fuentes A.M.-P., George N., Fexova S., Fonseca N.A., Füllgrabe A., Green M., Huang N. et al. Expression Atlas update: from tissues to single cells. Nucleic Acids Res. 2020; 48:D77–D83. 31665515 10.1093/nar/gkz947 PMC7145605

[21] Sondka Z., Bamford S., Cole C.G., Ward S.A., Dunham I., Forbes S.A. The COSMIC Cancer Gene Census: describing genetic dysfunction across all human cancers. Nat. Rev. Cancer. 2018; 18:696–705. 30293088 10.1038/s41568-018-0060-1 PMC6450507

[22] consortium T.E.P. Europe PMC: a full-text literature database for the life sciences and platform for innovation. Nucleic Acids Res. 2015; 43:D1042–D1048. 25378340 10.1093/nar/gku1061 PMC4383902

[23] Behan F.M., Iorio F., Picco G., Gonçalves E., Beaver C.M., Migliardi G., Santos R., Rao Y., Sassi F., Pinnelli M. et al. Prioritization of cancer therapeutic targets using CRISPR–Cas9 screens. Nature. 2019; 568:511–516. 30971826 10.1038/s41586-019-1103-9

[24] Buniello A., Jacqueline A., Cerezo M., Harris L.W., Hayhurst J., Malangone C., McMahon A., Morales J., Mountjoy E., Sollis E. et al. The NHGRI-EBI GWAS Catalog of published genome-wide association studies, targeted arrays and summary statistics 2019. Nucleic Acids Res. 2019; 47:D1005–D1012. 30445434 10.1093/nar/gky1120 PMC6323933

[25] Zhou W., Nielsen J.B., Fritsche L.G., Dey R., Gabrielsen M.E., Wolford B.N., Lefaive J., Vandehaar P., Gagliano S.A., Gifford A. et al. Efficiently controlling for case-control imbalance and sample relatedness in large-scale genetic association studies. Nat. Genet. 2018; 50:1335–1341. 30104761 10.1038/s41588-018-0184-y PMC6119127

[26] Shefchek K.A., Harris N.L., Gargano M., Matentzoglu N., Unni D., Brush M., Keith D., Conlin T., Vasilevsky N., Zhang X.A. et al. The monarch initiative in 2019: an integrative data and analytic platform connecting phenotypes to genotypes across species. Nucleic Acids Res. 2020; 48:D704–D715. 31701156 10.1093/nar/gkz997 PMC7056945

[27] Brown K.K., Hann M.M., Lakdawala A.S., Santos R., Thomas P.J., Todd K. Approaches to target tractability assessment – a practical perspective. MedChemComm. 2018; 9:606–613. 30108951 10.1039/c7md00633k PMC6072525

[28] Bowes J., Brown A.J., Hamon J., Jarolimek W., Sridhar A., Waldron G., Whitebread S. Reducing safety-related drug attrition: the use of in vitro pharmacological profiling. Nat. Rev. Drug Discov. 2012; 11:909–922. 23197038 10.1038/nrd3845

[29] Lynch J.J., Van Vleet T.R., Mittelstadt S.W., Blomme E.A.G. Potential functional and pathological side effects related to off-target pharmacological activity. 2017; 10.1016/j.vascn.2017.02.020 28216264

[30] Force T., Kolaja K.L. Cardiotoxicity of kinase inhibitors: the prediction and translation of preclinical models to clinical outcomes. Nat. Rev. Drug Discov. 2011; 10:111–126. 21283106 10.1038/nrd3252

[31] Lamore S.D., Ahlberg E., Boyer S., Lamb M.L., Hortigon-Vinagre M.P., Rodriguez V., Smith G.L., Sagemark J., Carlsson L., Bates S.M. et al. Deconvoluting kinase inhibitor induced cardiotoxicity. Toxicol. Sci. 2017; 158:213–226. 28453775 10.1093/toxsci/kfx082 PMC5837613

[32] Cases M., Briggs K., Steger-Hartmann T., Pognan F., Marc P., Kleinöder T., Schwab C., Pastor M., Wichard J., Sanz F. The eTOX data-sharing project to advance in silico drug-induced toxicity prediction. 2014; 15:21136–21154. 10.3390/ijms151121136 PMC4264217 25405742

[33] Krewski D., Acosta D., Andersen M., Anderson H., Bailar J.C., Boekelheide K., Brent R., Charnley G., Cheung V.G., Green S. et al. Toxicity testing in the 21st century: a vision and a strategy. J. Toxicol. Environ. Health B. 2010; 13:51–138. 10.1080/10937404.2010.483176 PMC4410863 20574894

[34] Carvalho-Silva D., Garcia L., Morgan S.L., Brooksbank C., Dunham I. Ten simple rules for delivering live distance training in bioinformatics across the globe using webinars. PLoS Comput. Biol. 2018; 14:e1006419. 30439935 10.1371/journal.pcbi.1006419 PMC6237289

[35] Nguyen D.-T., Mathias S., Bologa C., Brunak S., Fernandez N., Gaulton A., Hersey A., Holmes J., Jensen L.J., Karlsson A. et al. Pharos: collating protein information to shed light on the druggable genome. Nucleic Acids Res. 2017; 45:D995–D1002. 27903890 10.1093/nar/gkw1072 PMC5210555

[36] Piñero J., Ramírez-Anguita J.M., Saüch-Pitarch J., Ronzano F., Centeno E., Sanz F., Furlong L.I. The DisGeNET knowledge platform for disease genomics: 2019 update. Nucleic Acids Res. 2019; 48:D845–D855. 10.1093/nar/gkz1021 PMC7145631 31680165

[37] Coker E.A., Mitsopoulos C., Tym J.E., Komianou A., Kannas C., Patrizio E., Ozer B., Antolin A.A., Workman P. et al. canSAR: update to the cancer translational research and drug discovery knowledgebase. Nucleic Acids Res. 2019; 47:D917–D922. 30496479 10.1093/nar/gky1129 PMC6323893

[38] Zhang W., Zhang H., Yang H., Li M., Xie Z., Li W. Computational resources associating diseases with genotypes, phenotypes and exposures. Brief. Bioinform. 2019; 20:2098–2115. 30102366 10.1093/bib/bby071 PMC6954426

[39] Fang H., De Wolf H., Knezevic B., Burnham K.L., Osgood J., Sanniti A., Lledó Lara A., Kasela S., De Cesco S., Wegner J.K. et al. A genetics-led approach defines the drug target landscape of 30 immune-related traits. Nat. Genet. 2019; 51:1082–1091. 31253980 10.1038/s41588-019-0456-1 PMC7124888

[40] Picart-Armada S., Barrett S.J., Willé D.R., Perera-Lluna A., Gutteridge A., Dessailly B.H. Benchmarking network propagation methods for disease gene identification. PLoS Comput. Biol. 2019; 15:e1007276. 31479437 10.1371/journal.pcbi.1007276 PMC6743778

