# Supplementary material for: Open Targets Platform: supporting systematic drug–target identification and prioritisation
Source: Nucleic Acids Res. 2020 Nov 16;49(D1):D1302–10. doi: 10.1093/nar/gkaa1027 (PMC7779013; doi:10.1093/nar/gkaa1027)
Supplement: gkaa1027_Supplemental_File [file gkaa1027_supplemental_file.pdf]

Open Targets Platform: supporting systematic drug-target identification and prioritisation

Supplemental

AUTHOR LIST

David Ochoa[1,2,*], Andrew Hercules[1,2], Miguel Carmona[1,2], Daniel Suveges[1,2], Asier Gonzalez-Uriarte[1,2], Cinzia Malangone[1,2], Alfredo Miranda[1,2], Luca Fumis[1,2], Denise Carvalho-Silva[1,2], Michaela Spitzer[1,2], Jarrod Baker[1,2], Javier Ferrer[1,2], Arwa Raies[1,2], Olesya Razuvayevskaya[1,2], Adam Faulconbridge[1,2], Eirini Petsalaki[1,2], Prudence Mutowo[2,3], Sandra Machlitt-Northen[2,3], Gareth Peat[1,2], Elaine McAuley[1,2], Chuang Kee Ong[1,2], Edward Mountjoy[2,4], Maya Ghoussaini[2,4], Andrea Pierleoni[1,2], Eliseo Papa[2,5], Miguel Pignatelli[1,2], Gautier Koscielny[2,3], Mohd Karim[2,4], Jeremy Schwartzentruber[2,4], David G. Hulcoop[2,3], Ian Dunham[1,2,4], Ellen M. McDonagh[1,2,*]

[1] European Molecular Biology Laboratory, European Bioinformatics Institute (EMBL-EBI), Wellcome Genome Campus, Hinxton, Cambridgeshire CB10 1SD, UK

[2] Open Targets, Wellcome Genome Campus, Hinxton, Cambridgeshire CB10 1SD, UK

[3] GlaxoSmithKline plc, GSK Medicines Research Centre, Gunnels Wood Road, Stevenage, SG1 2NY, UK

[4] Wellcome Sanger Institute, Wellcome Genome Campus, Hinxton, Cambridgeshire CB10 1SA, UK

[5] Systems Biology, Biogen, Cambridge, MA, 02142, USA

* To whom correspondence should be addressed. Tel: 01223 494330. Email: emcdonagh@ebi.ac.uk. Present Address: Open Targets and European Molecular Biology Laboratory - European Bioinformatics Institute (EMBL-EBI), Wellcome Genome Campus, Hinxton, Cambridgeshire CB10 1SD, UK. Correspondence may also be addressed to David Ochoa, Tel: 01223 494330. Email: ochoa@ebi.ac.uk. Present Address: Open Targets and European Molecular Biology Laboratory - European Bioinformatics Institute (EMBL-EBI), Wellcome Genome Campus, Hinxton, Cambridgeshire CB10 1SD, UK.

Contents

Supplementary Figure 1: Evidence strings added per Open Targets release from each resource 3

Supplementary Table 1: Entities within the Open Targets Platform 4

Supplementary Table 2: Data sources used in the Open Targets Platform and weight for scoring of evidence 6

Supplementary Table 3: Github repositories 9

Supplementary Table 4: Availability / Outreach Activities links 11

References 12

Supplementary Figure 1: Evidence strings added per Open Targets release from each resource

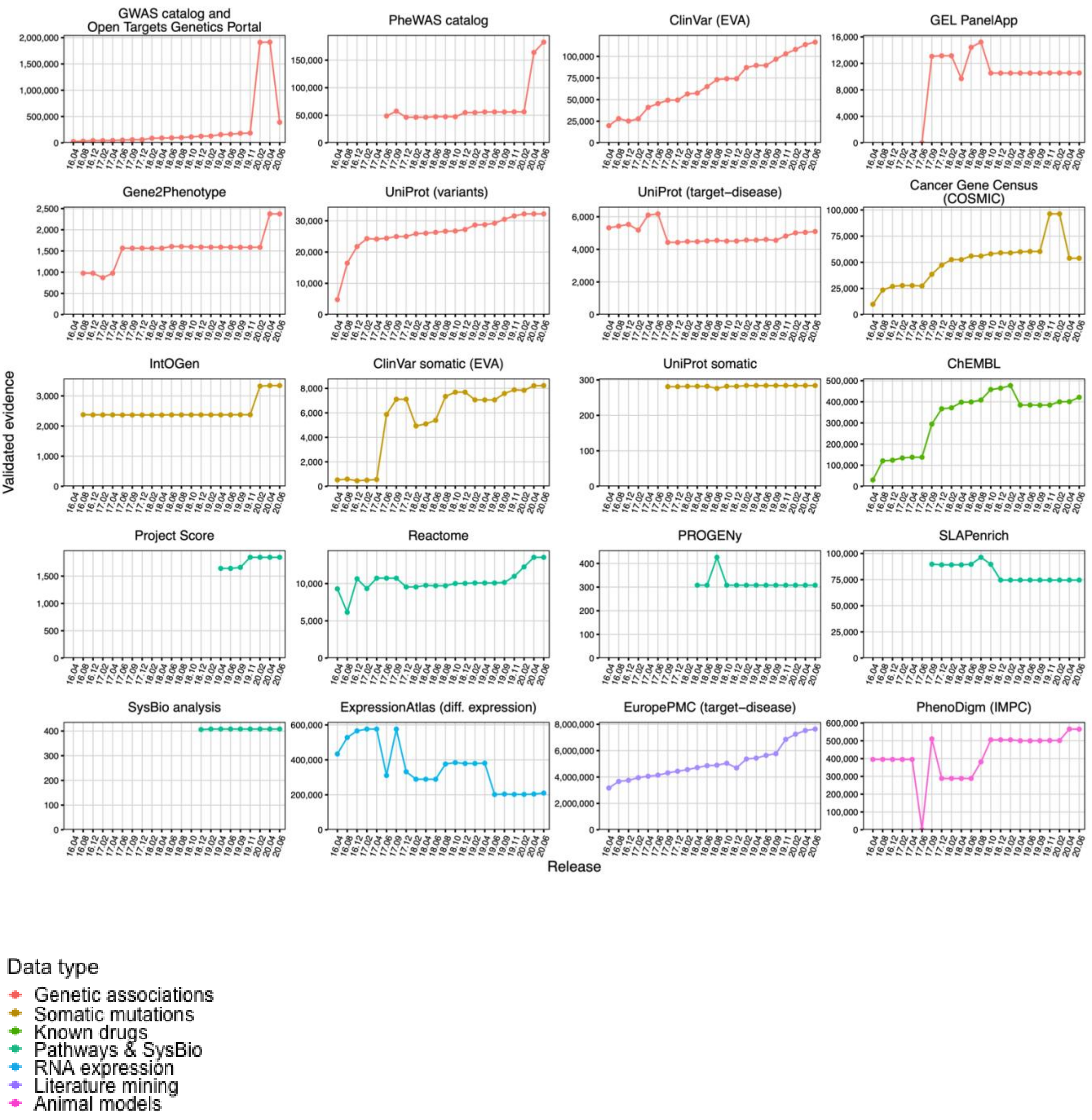

Supplementary Figure 1: The number of validated evidence strings integrated into each data release of the Open Target Targets Platform, for each data resource used for target-drug evidence generation.

Supplementary Table 1: Entities within the Open Targets Platform

Entity	Annotation	Source
Target	Protein Information<br>Functional annotation<br>Positional information<br>Structural information	Uniprot
Target	Protein Interactions	OmniPath DB
Target	Pathways	Reactome
Target	Baseline expression	Expression Atlas, GTEx, HPA
Target	Variants, Isoforms and Genomic Context	Ensembl
Target	Comparative genomics	Ensembl Compara
Target	Mouse Phenotypes	MGI
Target	Cancer Hallmarks	Cosmic / Cancer Gene Census
Target	Cancer Biomarkers	Cancer Genome Interpreter
Target	Chemical Probes	SGC, CP Portal, OS Probes
Target	Chemical Probes (predicted)	Probe Miner
Target	Bibliography	EuropePMC / LINK
Target	Target tractability	ChEMBL and others
Target	Target safety	HeCaTos, Tox21, eTOX and others
Target	Target Enabling Packages	SGC
Target	Drugs	ChEMBL, DailyMed, clinicaltrials.gov
Target	CRISPR-Cas9 cancer cell line dependency	Project Score
Target	Protein Structure	PDBe
Target	Gene Ontology	UniProt
Drug	Molecule information<br>Structure<br>Modality<br>Withdrawal<br>Mechanism of action	ChEMBL
Drug	Clinical Trial Information	Clinicaltrials.gov, DailyMed
Drug	Bibliography	EuropePMC / LINK
Drug	Pharmacovigilance	openFDA/FAERS
Disease	Ontology<br>- Cross-references<br>- Synonyms<br>- Classification	EFO

Disease	Phenotypes	EFO
Disease	Bibliography	EuropePMC / LINK
Disease	Drugs	ChEMBL, DailyMed, clinicaltrials.gov

Supplementary Table 2: Data sources used in the Open Targets Platform and weight for scoring of evidence

Data source	Score description	Weight factor	Source URL	Source Reference (if available)
OT Genetics Portal	Locus 2 gene (L2G) score, lower threshold: 0.05	1	https://genetics.opentargets.org/	
PheWAS Catalog	Functional consequence score of variants, normalised p-value and normalised sample size	1	https://phewascatalog.org/	1
EVA	Functional consequence score of variants e.g. germline variants that cause transcript ablation will have a score of 1, whereas variants that are intronic will have a score of 0.5	1	EVA: https://www.ebi.ac.uk/eva/?Home ClinVar: https://www.ncbi.nlm.nih.gov/clinvar/	2,3
Genomics England PanelApp	Gene-disease associations are curated and crowdsourced by experts and will have the highest score of 1	1	https://panelapp.genomicsengland.co.uk/	4
Gene2Phenotype	Gene-disease associations are inferred by curators and will have a score of 1, the highest functional consequence score	1	https://www.ebi.ac.uk/gene2phenotype	5
Uniprot Literature	Curator inference score based on how strong the evidence for the gene's involvement in the disease is. If the evidence is strong, the score will be 1. For evidence deemed not to be strong by the curator, the score will be 0.5	1	https://www.uniprot.org/	6
Uniprot	Functional consequence score of variants e.g. germline variants that cause transcript ablation will have a score of 1, whereas variants that are intronic will have a score of 0.5	1	https://www.uniprot.org/	6

ChEMBL	Clinical trials phase binned score. Scores will be 0.09 for phase 0, 0.1 for phase I, 0.2 for Phase II, 0.7 for Phase III, and 1 for Phase IV drugs	1	https://www.ebi.ac.uk/chembl/	7
Reactome	Functional consequence of 1 for a pathway inferred by a curator	1	https://reactome.org/	8
CRISPR	CRISPR evidence is scored as per the priority score described by Behan et al. 2019 (this originally varies from 0 to 100 and is available in Table 6 as supplementary information; any value above 40 is significant) divided by 100	1	https://score.depmap.sanger.ac.uk/	9
SLAPenrich	Scored according to Iorio F et al 2018, followed by quantifying, in large cohorts of cancer patients, the divergence of the total number of samples with genomic alterations in pathway from its expectation, accounting for mutational burdens and total exonic block lengths of genes in that pathway	0.5	https://saezlab.github.io/SLAPenrich/	10
SysBio	p-values or rank-based scores are used for scoring if provided, otherwise a score of 0.5 is assigned	0.5	NA	NA
PROGENy	Scored per sample and pathway following a modification of the original implementation described in the reference.	0.5	https://saezlab.github.io/progeny/	11
Expression Atlas	Normalised p-value, normalised expression fold change and normalised percentile rank	0.2	https://www.ebi.ac.uk/gxa/home	12
Cancer Gene Census	Base score of 0.5 modified as follows: -0.25 if only 1 mutated sample, +0.25 if gene Tier 1 and mutated more frequently in particular disease compared to all other diseases and +0.25 if gene Tier 1 and mutations occur more frequently than in other genes of similar length in the same disease	1	https://cancer.sanger.ac.uk/census	13
intOGen	Combined q-value of driver identification methods	1	www.intogen.org/search	14
Uniprot somatic	Curator inference score based on how strong the evidence for the gene's involvement in the disease is. If the evidence is strong, the score will be 1. For evidence deemed not to be strong by the curator, the score will be 0.5	1	https://www.uniprot.org/	6

EVA somatic	Functional consequence score of variants e.g. germline variants that cause transcript ablation will have a score of 1, whereas variants that are intronic will have a score of 0.5	1	EVA: https://www.ebi.ac.uk/eva/?Home ClinVar: https://www.ncbi.nlm.nih.gov/clinvar/	2,3
Europe PMC	Weighted document sections, sentence locations and title for full text articles and abstracts	0.2	http://europepmc.org/	15,16
PhenoDigm	Similarity score between a mouse model and a human disease described in the reference	0.2	https://www.sanger.ac.uk/tool/phenodigm/	17

Supplementary Table 3: Github repositories

**a) Evidence file generation**	
Repository https://github.com/opentargets/...	Description
evidence_datasource_parsers	Python scripts to generate evidence strings from csv or text files for:<br>- Project Score (aka CRISPR)<br>- Gene2Phenotype<br>- OT Genetics Portal<br>- Genomics England Panel App<br>- IntOGen<br>- IMPC/PhenoDigm (aka MouseModels)<br>- PROGENy<br>- PheWAS catalg<br>- SLAPenrich<br>- Systems Biology
**b) Data ingest and analysis**	
Repository https://github.com/opentargets/...	Description
platform-input-support	Application that ensures reproducibility of data release by copying input files into a specific google storage bucket and generating a YAML config file used to run the pipeline
data_pipeline	ExtracTransform-Load (ETL) pipeline that processes all the data files and generates the elasticsearch indices used by the web app
library-beam	ETL pipeline for NLP analysis of Medline and PubMed to annotate publications of targets and diseases
**c) Infrastructure, API and web application**	
Repository https://github.com/opentargets/...	Description
webapp	Angular.js web application
rest_api	Flask REST API for Open Targets Platform

library-api	REST API to serve data generated by Open Targets Library
**d) Other**	
Repository https://github.com/opentargets/...	Description
json_schema	JSON schema for evidence files
validator	Python evidence file validator
opentargets-py	Python client for the Open Targets REST API
expression_analysis	The rna_expression_analysis_with_blueprint2.ipynb notebook contains the python code used to process the baseline expression meta-analysis file
**e) External repositories**	
URL	Description
https://github.com/EBIvariation/eva-opentargets	EVA pipeline to generate evidence from ClinVar dumps
https://github.com/EBISPOT/efo	Experimental Factor Ontology
https://github.com/ebi-uniprot/open-targets-core-db	UniProt pipeline to generate evidence
https://github.com/reactome/data-export	Module to export files based on queries to the Reactome Graph database
https://github.com/suhaibMo/BaselineMetaAnalysis	Scripts to perform meta-analysis of several baseline expression datasets
https://github.com/melschneider/tractability_pipeline_v2	Pipeline that generates small molecule, antibody, and other modality tractability assessments

Supplementary Table 4: Availability / Outreach Activities links

URL	Description
http://blog.opentargets.org/	The Open Targets blog, which includes release posts and in-depth articles on technical and scientific aspects of the Platform and Open Targets more broadly
https://www.targetvalidation.org/outreach	Listing of all previous and upcoming Open Targets Platform training workshops and webinars
https://docs.targetvalidation.org/	Homepage for all Open Targets Platform documentation
https://docs.targetvalidation.org/programmatic-access/rest-api	REST API documentation
https://opentargets.readthedocs.io/en/stable/	Python client documentation
https://www.targetvalidation.org/downloads/data	Page with links to all data files available for download using Google Cloud storage
ftp://ftp.ebi.ac.uk/pub/databases/opentargets/platform/	EMBL-EBI FTP service that hosts the input and output files
https://docs.targetvalidation.org/release-notes	Release notes for each release
https://docs.targetvalidation.org/technical-pipeline/technical-notes	Technical notes for each release
https://github.com/opentargets	Open Targets GitHub organisation page listing all repositories

References

1. Denny, J. C. *et al.* Systematic comparison of phenome-wide association study of electronic medical record data and genome-wide association study data. *Nat. Biotechnol.* **31**, 1102–1110 (2013).
2. Cook, C. E. *et al.* The European Bioinformatics Institute in 2016: Data growth and integration. *Nucleic Acids Res.* **44**, D20-26 (2016).
3. Landrum, M. J. *et al.* ClinVar: improving access to variant interpretations and supporting evidence. *Nucleic Acids Res.* **46**, D1062–D1067 (2018).
4. Martin, A. R. *et al.* PanelApp crowdsources expert knowledge to establish consensus diagnostic gene panels. *Nat. Genet.* **51**, 1560–1565 (2019).
5. Thormann, A. *et al.* Flexible and scalable diagnostic filtering of genomic variants using G2P with Ensembl VEP. *Nat. Commun.* **10**, 2373 (2019).
6. UniProt Consortium. UniProt: a worldwide hub of protein knowledge. *Nucleic Acids Res.* **47**, D506–D515 (2019).
7. Mendez, D. *et al.* ChEMBL: towards direct deposition of bioassay data. *Nucleic Acids Res.* **47**, D930–D940 (2019).
8. Jassal, B. *et al.* The reactome pathway knowledgebase. *Nucleic Acids Res.* **48**, D498–D503 (2020).
9. Behan, F. M. *et al.* Prioritization of cancer therapeutic targets using CRISPR-Cas9 screens. *Nature* **568**, 511–516 (2019).
10. Iorio, F. *et al.* Pathway-based dissection of the genomic heterogeneity of cancer hallmarks' acquisition with SLAPenrich. *Sci. Rep.* **8**, 6713 (2018).
11. Schubert, M. *et al.* Perturbation-response genes reveal signaling footprints in cancer gene expression. *Nat. Commun.* **9**, 20 (2018).
12. Papatheodorou, I. *et al.* Expression Atlas update: from tissues to single cells. *Nucleic Acids Res.* **48**, D77–D83 (2020).
13. Sondka, Z. *et al.* The COSMIC Cancer Gene Census: describing genetic dysfunction across all human cancers. *Nat. Rev. Cancer* **18**, 696–705 (2018).
14. Martínez-Jiménez, F. *et al.* A compendium of mutational cancer driver genes. *Nat. Rev. Cancer* (2020) doi:10.1038/s41568-020-0290-x.
15. Europe PMC Consortium. Europe PMC: a full-text literature database for the life sciences and platform for innovation. *Nucleic Acids Res.* **43**, D1042-1048 (2015).
16. Kafkas, Ş., Dunham, I. & McEntyre, J. Literature evidence in open targets - a target validation platform. *J. Biomed. Semant.* **8**, 20 (2017).
17. Smedley, D. *et al.* PhenoDigm: analyzing curated annotations to associate animal models with human diseases. *Database J. Biol. Databases Curation* **2013**, bat025 (2013).
